# Psychometric and diagnostic properties of the Taiwan version of the Quick Mild Cognitive Impairment screen

**DOI:** 10.1371/journal.pone.0207851

**Published:** 2018-12-03

**Authors:** Meng-Ta Lee, Wan-Ying Chang, Yuh Jang

**Affiliations:** 1 School of Occupational Therapy, College of Medicine, National Taiwan University, Taipei, Taiwan; 2 Division of Occupational Therapy, Taipei Hospital, Ministry of Health and Welfare, New Taipei, Taiwan; 3 Department of Physical Medicine and Rehabilitation, National Taiwan University Hospital, Taipei, Taiwan; University of Wuerzburg, GERMANY

## Abstract

There is a need for a screening tool with capacities of accurate detection of early mild cognitive impairment (MCI) and dementia and is suitable for use in a range of languages and cultural contexts. This research aims to evaluate the psychometric and diagnostic properties of the Taiwan version of Q*mci* (Q*mci*-TW) screen and to explore the discriminating ability of the Q*mci*-TW in differentiating among normal controls (NCs), MCI and dementia. Thirty-one participants with dementia and 36 with MCI and 35 NCs were recruited from a neurology department of regional hospital in Taiwan. Their results on the Q*mci*-TW, Taiwanese version of the Montreal Cognitive Assessment (MoCA), and Traditional Chinese version of the Mini–Mental State Examination (MMSE) were compared. For analysis, we used Cronbach’s *α*, intraclass correlation coefficient, Spearman’s *ρ*, Kruskal–Wallis test, receiver operating characteristic curve analysis, and multivariate analysis, as appropriate. The Q*mci*-TW exhibited satisfactory test–retest reliability, internal consistency, and interrater reliability as well as a strong positive correlation with results from the MoCA and MMSE. The optimal cut-off score on the Q*mci*-TW for differentiating MCI from NC was ≤ 51.5/100 and dementia from MCI was ≤ 31/100. The MoCA exhibited the highest accuracy in differentiating MCI from NC, followed by the Q*mci*-TW and then MMSE; whereas, the Q*mci*-TW and MMSE exhibited the same accuracy in differentiating dementia from MCI, followed by the MoCA. The Q*mci*-TW may be a useful clinical screening tool for a spectrum of cognitive impairments.

## Introduction

The number of people aged older than 65 years is increasing worldwide [1]. The population of older people with dementia is expected to increase concurrently with global aging [2, 3]. The reported proportion of individuals with mild cognitive impairment (MCI) is expected even higher than of those with dementia [4, 5]; however, in clinical practice, most MCI cases in older adults remain unidentified. Although people with MCI are completely capable of self-care activities, they exhibit slight impairment in memory, attention, orientation to time, visuospatial perception, problem solving, instrumental activities of daily living, and judgment; these impairments may affect community participation and job security [6, 7]. Individuals with MCI involving instrumental activities of daily living limitations are more likely to develop dementia [7]. In general, people with MCI are at a greater risk of developing dementia [8] than other aged-matched individuals [9], and approximately half of progressions into dementia occur within a 5-year period [10]. Differentiation among individuals with MCI and dementia and normal controls (NCs) is critical for appropriate pharmacotherapeutic and nonpharmacotherapeutic treatment courses [11, 12].

In Taiwan, the most widely used cognitive screening instruments are the Traditional Chinese version of the Mini–Mental State Examination (MMSE) [13] and the Taiwanese version of the Montreal Cognitive Assessment (MoCA) [14]. On comparing the MMSE and MoCA, the MoCA can efficiently discriminate MCI, whereas the MMSE can efficiently discriminate dementia [15]. The ceiling effect and lack of dynamic performance range in the MMSE increase the likelihood that individuals in the early stages of MCI or dementia score within the normal range [16]. Moreover, half of the MoCA subtests have shown significant floor effects in assessments of Parkinson’s disease [17]. Floor and ceiling effects must be considered in cognitive function tests. Cognitive screening tools with appropriate diagnostic properties are critical for effective clinical practice.

The Quick Mild Cognitive Impairment (Q*mci*) screen is a brief cognitive screening instrument, validated for use in Australian, Canadian, Dutch, Irish, and Turkish populations [18–22]. The Q*mci*, developed through the AB Cognitive Screen 135 [23], added logical memory and reweights the scores of all subtests for maximizing sensitivity and specificity for differentiating NCs from MCI. The Q*mci* is more accurate in differentiating individuals with MCI from NCs than the standardized MMSE [24]; it also has similar accuracy to and greater specificity than the MoCA [18–20]. The Q*mci* is strongly associated with the Clinical Dementia Rating scale, Alzheimer’s Disease Assessment Scale-Cognitive Section and Lawton-Brody IADL scale [24]. The overview of the Q*mci*, MMSE, and MoCA are presented in S1 Table.

In busy clinical settings, the Q*mci* is superior to the MMSE and MoCA with similar reliability, less administered time and better diagnostic properties for the purpose of early detection and treatment. Thus, because the Q*mci* is useful for detecting early MCI and variations in cognitive function between NCs and those with severe dementia, a Taiwan version is required. The research questions are (1) whether the Taiwan version of the Q*mci* (Q*mci*-TW) screen yields sound psychometric properties (reliability and validity) and diagnostic properties (sensitivity, specificity, and predictive values) in Taiwan population, and (2) whether the Q*mci*-TW exhibits satisfactory discriminating ability to differentiate NCs from MCI and/or dementia from MCI. This study aims to assess the psychometric and diagnostic properties of the Q*mci*-TW screen and to explore the discriminating ability of the Q*mci*-TW in differentiating among NCs, MCI and dementia.

## Materials and methods

### Participants

A power calculation based on published data of the Q*mci* related studies was performed using the PAIRSetc.EXE program of the WinPepi software [25, 26]. We used the McNemar’s test to examine the differences of the area under the curve (AUC) between the Q*mci* and MMSE, and the Q*mci* and MoCA for matched samples to estimate the required sample sizes. The results indicated that the study would require minimum 80 paired observations to demonstrate significant difference between the instruments and their ability to differentiate MCI from NCs, at a significance level of 0.05 and power of 80% [20, 21].

Participants were categorized into three groups: MCI, dementia, and NCs. Between May 2017 and December 2017, participants were recruited consecutively from the neurology outpatient department of regional hospital in New Taipei, Taiwan. NCs without subjective and objective cognitive problems were recruited from convenience sampling. Only the individuals aged ≥ 65 years and able to follow instructions and understand the content of the assessments through verbal communication were eligible for participation. Dementia (Alzheimer’s disease or vascular or mixed dementia subtypes) and amnestic-type MCI were diagnosed according to the Diagnostic and Statistical Manual of Mental Disorders, Fourth Edition [27] and the National Institute of Neurological and Communicative Diseases and Stroke and the Alzheimer's Disease and Related Disorders Association [28] criteria and National Institute on Aging-Alzheimer’s Association workgroup’s diagnostic guidelines for Alzheimer’s disease [29], as applicable. The participants with MCI and dementia were classified using the Clinical Dementia Rating scale global scores of 0.5 and 1–3, respectively [6]. Participants were excluded if they scored > 7 on the Geriatric Depression Scale-Short Form, indicating depression status [30]; or were diagnosed with other MCI or dementia subtypes, including frontotemporal dementia, Parkinson’s disease, or Lewy Body dementia, that present infrequently, typically with exaggerated functional deficits and different MCI syndromes.

The study protocol for the protection of human participants and the consent procedure was approved by the Institutional Review Board of the Taipei Hospital, Ministry of Health and Welfare (TH-IRB-0016-0033). Before the study, the purpose and procedure of the research were explained to all participants. NCs signed informed consents by themselves; whereas, participants with MCI or dementia signed informed consents along with their legal guardians. The participants’ background information was protected as confidential and was used only for research purposes.

### Data collection

In this cross-sectional study, the demographic data, including age in years, gender, and years of education, were collected. The same trained rater, blinded to final diagnosis, alternately and randomly administered the Q*mci*-TW, MoCA, MMSE, Barthel Index [31] and Lawton Instrumental Activities of Daily Living scale [32] on the same day. Additionally, after 2 weeks, the Q*mci*-TW, with alternative versions [33], was readministered to randomly selected participants by the two trained raters, blinded to final diagnosis.

In total, 119 participants were recruited, but 17 participants who scored > 7 on the Geriatric Depression Scale-Short Form were excluded. The remaining 102 participants (47 men and 55 women) were included for further study. Among the final sampled patients, 35 (34.3%) were NCs, 36 (35.3%) had received a MCI diagnosis, and 31 (30.4%) had received a diagnosis of dementia. Participants with dementia, classified using Clinical Dementia Rating scale global scores, were grouped according to mild (*n* = 12), moderate (*n* = 13), and severe dementia (*n* = 6). The NCs (*p* < 0.001) and participants with MCI (*p* = 0.005) were significantly younger than those with dementia. NCs had received significantly more education than those with MCI (*p* = 0.007) or dementia (*p* < 0.001). The mean Geriatric Depression Scale-Short Form scores among NCs was significantly lower than that among participants with dementia (*p* < 0.001). The demographic characteristics of the participants are presented in Table 1.

**Table 1:** The demographic characteristics of the participants stratified by NC, MCI and dementia.

Characteristics	Total (*n* = 102)	NC (*n* = 35)	MCI (*n* = 36)	Dementia (*n* = 31)	*χ*^*2*^ (*df* = 2)	Pairwise comparison^a^
Age in years	77.13 ± 7.49	73.64 ± 6.39	76.22 ± 7.41	82.11 ± 6.13	22.91**	3 > 1, 3 > 2
Gender [Male, Number (%)]	47 (46.1%)	17 (48.6%)	14 (38.9%)	16 (51.6%)	1.22	
Years in education	7.26 ± 4.87	10.03 ± 3.85	6.83 ± 4.87	4.61 ± 4.35	23.48**	1 > 2, 1 > 3
GDS-SF	2.75 ± 1.93	1.83 ± 1.72	2.69 ± 1.62	3.84 ± 1.97	17.48**	3 > 1

The listed statistics were mean ± standard deviation (SD) or frequency (percentage).

**Abbreviations:** NC, Normal control; MCI, Mild cognitive impairment; and GDS-SF, Geriatric Depression Scale-Short Form.

^a^1. Normal control group; 2. Mild cognitive impairment group; and 3. Dementia group.

*p < 0.05.

**p < 0.001.

### Instruments

The Q*mci*-TW is a performance test, containing six subtests, namely orientation, registration, clock drawing, delayed recall, verbal fluency, and logical memory. The Q*mci* can be administered and scored with a median time of under 5 minutes, and the alternative word groups and versions of the registration and recall task and the verbal fluency and logical memory subtests in the Q*mci* have been validated for convenience [33]. We translated the Q*mci* into the Q*mci*-TW following the established translation guidelines [34]. The Q*mci*-TW was administered and scored using the test manual’s instructions. The Q*mci*-TW scores ranged from 0 to 100, with a higher score indicating greater cognitive function.

The MMSE is also a performance test, standardized and validated to measure cognitive functions in orientation, registration, attention, calculation, recall, language, and copying. MMSE scores range from 0 to 30, with a higher score indicating greater cognitive function [13].

The MoCA is a standardized and validated tool designed to measure cognitive functions in visuospatial and executive tasks, naming, memory, attention, language, abstraction, delayed recall, and orientation. In MoCA scoring, 1 point is added for individuals whose educational level is ≤ 12, with scores ranging from 0 to 30 and a higher score indicating greater cognitive function [14].

The Barthel Index is a validated tool designed to measure activities of daily living independence, specifically regarding feeding, bathing, grooming, dressing, bowels, bladder, toilet use, transfers, mobility, and stairs. Barthel Index scores range from 0 to 20, with a higher score indicating greater independence [31].

The Lawton Instrumental Activities of Daily Living scale is a validated tool designed to measure instrumental activities of daily living independence, specifically with regard to telephone use, shopping, food preparation, housekeeping, laundry, transport mode, medication responsibility, and finance-handling ability. The scores range from 0 to 8, with a higher score indicating greater independence in complex activities of daily living [32].

### Statistical analysis

Statistical analysis was performed using the IBM SPSS Statistics software, version 19.0 (IBM Corporation, Somers, NY, U.S.A.) and the R 3.4.3 software (R Foundation for Statistical Computing, Vienna, Austria). The Shapiro-Wilk test was used to test for normality, and the results indicated that most data were nonparametric. The distributional properties of continuous variables are presented as means ± standard deviations and categorical variables as frequencies and percentages. Missing values were given zero score following the manuals and scoring guidelines of each assessments. For the Q*mci*-TW, we used Cronbach’s *α*, intraclass correlation coefficients (ICCs), and Spearman’s *ρ* for internal consistency, test–retest and interrater reliability, and concurrent and construct validity [35], respectively. Data were analysed using Kruskal–Wallis test and *post hoc* Dunn’s test, and receiver operating characteristic (ROC) curves for between-group comparisons, and AUC [35], respectively.

#### Floor and ceiling effects

Frequency was used to calculate the lowest and highest raw scores for a subtest as the floor and ceiling, respectively. Floor and ceiling effects were considered significant if they were exhibited in more than 20% of the sample [36]. The ceiling and floor effects indicated that a subtest was too easy and too difficult for the study population, respectively. A subtest with ceiling or floor effects is considered non-sensitive, and thus, unsuitable for use in discriminating between groups [37].

#### Internal consistency

Cronbach’s *α* coefficient was used to examine the internal consistency of the Q*mci*-TW screen subtests. This coefficient estimates the reliability of an instrument according to the consistency of the subtests, accounting for the number of subtests. Cronbach’s *α* > 0.7 was considered acceptable for internal consistency [37]. Inter-item correlation was also used to examine the correlations between the subtests of the Q*mci*-TW screen.

#### Test–retest reliability and interrater reliability

The ICCs were used to examine the test-retest and interrater reliability of the Q*mci*-TW screen. The ICCs of 0.75–1.00 indicated an excellent reliability [38].

#### Concurrent and construct validity

The Spearman’s correlation coefficients were estimated between the Q*mci*-TW screen and the other validated cognitive screening instruments, MoCA and MMSE, to determine concurrent validity, and between Q*mci*-TW and the validated activities of daily living and instrumental activities of daily living assessments, Barthel Index and Lawton Instrumental Activities of Daily Living scale, to determine construct validity. The Spearman’s *ρ* of 0.4–0.8 indicated an acceptable validity [39].

#### Sensitivity, specificity, and predictive values

The ROC curve analysis was used to calculate diagnostic accuracy based on the AUC. The AUC ≥ 0.8 and ≥ 0.9 represented good and excellent discriminating powers respectively. The optimal cut-off scores were derived using Youden’s Index [40]. Sensitivity, specificity, positive predictive value (PPV), and negative predictive value (NPV) were calculated based on optimal cut-off scores.

To compare the predictive power among the three key instruments, Q*mci*-TW, MMSE, and MoCA, multivariate analysis was conducted by fitting two logistic regression models of (1) MCI or dementia *versus* NC in all subjects (*n* = 102) and (2) dementia *versus* MCI in the subjects with MCI or dementia (*n* = 67). The goal of regression analysis was to find one or a few parsimonious regression models that fitted the observed data well for effect estimation and/or outcome prediction. To ensure a good quality of analysis, the model-fitting techniques for (1) variable selection, (2) goodness-of-fit assessment, and (3) regression diagnostics and remedies were used in our logistic regression analyses. Specifically, the stepwise variable selection procedure (with iterations between the forward and backward steps) was applied to obtain the best candidate final logistic regression model using the My.stepwise package of R [41]. As listed in Tables 1 and 2, all the univariate significant and non-significant covariates, including age, gender, education level, and so on, were put on the variable list to be selected. Simple and multiple generalized additive models were fitted to detect nonlinear effects of continuous covariates and identify appropriate cut-off points for discretizing continuous covariates, if necessary, during the stepwise variable selection procedure. The significance levels for entry and for stay were set to 0.15 for being conservative. Then, with the aid of substantive knowledge, the best candidate final logistic regression model was identified manually by dropping the covariates with *p* value > 0.05 one at a time until all regression coefficients were significantly different from 0. Moreover, the goodness-of-fit measure, the estimated area under the ROC curve (also called the *c* statistic), and the Hosmer-Lemeshow goodness-of-fit test were examined to assess the goodness-of-fit of the fitted logistic regression model. Finally, the statistical tools of regression diagnostics for residual analysis, detection of influential cases, and check of multicollinearity were applied to discover any model or data problems.

**Table 2:** The clinical characteristics of the participants stratified by NC, MCI and dementia.

Characteristics	Total (n = 102)	NC (n = 35)	MCI (n = 36)	Dementia (n = 31)	*χ*^*2*^ (*df* = 2)	Pairwise comparison ^a^
**Q*mci*-TW**	42.80 ± 21.84	64.06 ± 8.43	43.13 ± 14.71	18.44 ± 11.48	71.19**	1 > 2 > 3
Orientation	7.79 ± 2.83	9.97 ± 0.17	8.39 ± 1.90	4.65 ± 2.54	62.32**	1 > 2 > 3
Registration	3.58 ± 1.21	4.49 ± 0.56	3.64 ± 0.72	2.48 ± 1.31	49.01**	1 > 2 > 3
Clock drawing	8.87 ± 5.59	13.14 ± 2.80	9.17 ± 4.77	3.71 ± 4.56	47.42**	1 > 2 > 3
Delayed recall	10.16 ± 8.01	17.49 ± 3.08	10.00 ± 7.06	2.06 ± 4.11	57.58**	1 > 2 > 3
Verbal fluency	6.20 ± 2.76	8.37 ± 2.07	6.38 ± 1.94	3.53 ± 1.89	53.25**	1 > 2 > 3
Logical memory	6.20 ± 5.12	10.57 ± 4.27	5.56 ± 4.23	2.00 ± 2.37	52.19**	1 > 2 > 3
**MoCA**	16.75 ± 7.81	24.51 ± 2.47	16.61 ± 5.05	8.13 ± 4.65	70.04**	1 > 2 > 3
Visuospatial and executive function	2.58 ± 1.60	3.91 ± 1.20	2.50 ± 1.34	1.16 ± 0.86	49.10**	1 > 2 > 3
Naming	1.90 ± 1.17	2.71 ± 0.52	1.92 ± 1.08	0.97 ± 1.11	35.09**	1 > 2 > 3
Memory	1.55 ± 0.61	1.89 ± 0.32	1.61 ± 0.55	1.10 ± 0.65	28.68**	1 > 3, 2 > 3
Attention	0.65 ± 0.48	1.00 ± 0.00	0.69 ± 0.47	0.19 ± 0.40	46.90**	1 > 2 > 3
Calculation	2.23 ± 0.98	2.97 ± 0.17	2.33 ± 0.72	1.26 ± 1.00	50.42**	1 > 2 > 3
Repetition	0.77 ± 0.80	1.37 ± 0.73	0.67 ± 0.72	0.23 ± 0.43	34.45**	1 > 2, 1 > 3
Verbal fluency	0.48 ± 0.50	0.94 ± 0.24	0.39 ± 0.49	0.06 ± 0.25	52.16**	1 > 2 > 3
Abstraction	0.37 ± 0.60	0.83 ± 0.71	0.19 ± 0.40	0.06 ± 0.25	31.95**	1 > 2, 1 > 3
Delayed recall	1.06 ± 1.41	2.23 ± 1.42	0.78 ± 1.17	0.06 ± 0.25	44.63**	1 > 2 > 3
Orientation	4.32 ± 2.05	5.91 ± 0.28	4.69 ± 1.49	2.10 ± 1.78	59.16**	1 > 2, 1 > 3
**MMSE**	22.68 ± 6.36	28.29 ± 1.18	23.61 ± 4.02	15.26 ± 4.58	75.20**	1 > 2 > 3
Orientation to time	3.57 ± 1.73	4.91 ± 0.28	3.94 ± 1.15	1.61 ± 1.48	61.08**	1 > 2 > 3
Orientation to place	3.83 ± 1.61	4.94 ± 0.24	4.17 ± 1.21	2.19 ± 1.60	58.35**	1 > 2 > 3
Registration	2.98 ± 0.14	3.00 ± 0.00	2.97 ± 0.17	2.97 ± 0.18	1.07	
Calculation	3.28 ± 1.78	4.66 ± 0.54	3.42 ± 1.46	1.58 ± 1.61	45.70**	1 > 2 > 3
Recall	1.42 ± 1.14	2.03 ± 0.89	1.47 ± 1.11	0.68 ± 1.01	23.49**	1 > 3, 2 > 3
Naming	1.98 ± 0.14	2.00 ± 0.00	2.00 ± 0.00	1.94 ± 0.25	4.63	
Repetition	0.91 ± 0.29	1.00 ± 0.00	0.92 ± 0.28	0.81 ± 0.40	7.60*	1 > 3
Reading comprehension	0.76 ± 0.43	1.00 ± 0.00	0.81 ± 0.40	0.45 ± 0.51	27.72**	1 > 3, 2 > 3
Writing	0.67 ± 0.47	1.00 ± 0.00	0.69 ± 0.47	0.26 ± 0.45	40.51**	1 > 2 > 3
Verbal comprehension and executive function	2.80 ± 0.58	3.00 ± 0.00	2.78 ± 0.59	2.61 ± 0.80	8.34*	1 > 3
Construction	0.48 ± 0.50	0.74 ± 0.44	0.50 ± 0.51	0.16 ± 0.37	22.14**	1 > 3, 2 > 3
**Barthel Index**	19.25 ± 2.32	19.89 ± 0.47	19.94 ± 0.33	17.74 ± 3.79	27.32**	1 > 3, 2 > 3
**Lawton IADL scale**	5.82 ± 2.41	7.54 ± 0.89	6.69 ± 1.28	2.87 ± 1.84	64.90**	1 > 3, 2 > 3

The listed statistics were mean ± standard deviation (SD).

**Abbreviations:** NC, Normal control; MCI, Mild cognitive impairment; Q*mci*-TW, Taiwan version of the Quick Mild Cognitive Impairment screen; MoCA, Montreal Cognitive Assessment; MMSE, Mini-Mental State Examination; and Lawton IADL scale, Lawton Instrument Activities of Daily Living scale.

^a^1: Normal control group; 2: Mild cognitive impairment group; and 3: Dementia group.

*p < 0.05.

**p < 0.001.

## Results

The total and most subtests of the Q*mci*-TW, MoCA, and MMSE scores of the NCs were significantly higher than those of participants with MCI and dementia groups, except for memory of the MoCA and registration, recall, naming, repetition, reading comprehension, verbal and executive function, and construction of the MMSE. Moreover, most of these scores were significantly higher in the MCI group than in the dementia group, except for repetition, abstraction, and orientation of the MoCA and registration, naming, repetition, and verbal and executive function of the MMSE. The Barthel Index and Lawton Instrumental Activities of Daily Living scale scores of the NCs and participants with MCI were significantly higher than those of participants with dementia (all *p*’s < 0.001). The clinical characteristics of the participants are presented in Table 2.

Although some of the demographic and clinical characteristics, including age, educational level, Geriatric Depression Scale-Short Form score, and so on, were significantly different among the subjects of NC, MCI, and dementia (Tables 1 and 2), logistic regression analysis of MCI or dementia *versus* NC in all subjects (*n* = 102) revealed that after the keen competitions during the stepwise variable selection procedure, the MoCA score, food preparation score of the Lawton IADL scale in the past, and calculation score of the MMSE stayed in the final logistic regression model as the most important statistically significant predictors (Table 3). Both the estimated area under the ROC curve = 0.99 and the modified Hosmer and Lemeshow goodness-of-fit *F* test *p* = 0.7479 (*df* = 9, 92) indicated an excellent fit.

**Table 3:** Multivariate analysis of the predictors of MCI or dementia *versus* NC among all subjects by fitting multiple logistic regression model with the stepwise variable selection method.

Covariate	Estimated Regression Coefficient	Estimated Standard Error	*z* Value	*p* Value	Estimated Odds Ratio	95% C.I. of Odds Ratio
Intercept	30.069	9.211	3.26	0.0011	–	–
MoCA score (0, 1, …, 30)	-0.817	0.240	-3.40	0.0007	0.4416	0.276–0.707
Food preparation score of the Lawton IADL scale in the past (0, 1)	-4.798	1.608	-2.98	0.0028	0.0082	< 0.001–0.193
Calculation score of the MMSE (0, 1, …, 5)	-1.986	0.953	-2.08	0.0371	0.1372	0.021–0.888

**Abbreviations:** NC, Normal control; MCI, Mild cognitive impairment; MoCA, Montreal Cognitive Assessment; MMSE, Mini-Mental State Examination; Lawton IADL scale, Lawton Instrument Activities of Daily Living scale; and 95% C.I., 95% Confidence Interval.

**Goodness-of-fit assessment:** *n* = 102, the estimated area under the Receiver Operating Characteristic (ROC) curve = 0.99 > 0.7, and the modified Hosmer and Lemeshow goodness-of-fit *F* test *p* = 0.7479 > 0.05 (*df* = 9, 92), which indicated an excellent fit.

**Prediction:** To calculate the estimated probability of being MCI or dementia (i.e., the *predicted value*, ${\hat{P}}_{i}$) given the observed covariate values, we can use the following formula. According to the above fitted multiple logistic regression model
$$\begin{array}{l} \mathrm{logit}\left({\hat{P}}_{i}\right)=\log\left(\frac{{\hat{P}}_{i}}{1‑{\hat{P}}_{i}}\right)\\ \;=30.069‑0.817\times (\mathrm{MoCA}\;\mathrm{score})‑4.798\times (\mathrm{food}\;\mathrm{preparation}\;\mathrm{score}\;\mathrm{of}\;\mathrm{the}\;\mathrm{Lawton}\;\mathrm{IADL}\;\mathrm{scale}\;\mathrm{in}\;\mathrm{the}\;\mathrm{past})‑1.986\times (\mathrm{calculation}\;\mathrm{score}\;\mathrm{of}\;\mathrm{the}\;\mathrm{MMSE}) \end{array}$$
the predicted value of observation *i* is
$${\hat{P}}_{i}=\frac{1}{1+\exp\{‑[30.069‑0.817\times (\mathrm{MoCA}\;\mathrm{score})‑4.798\times (\mathrm{food}\;\mathrm{preparation}\;\mathrm{score}\;\mathrm{of}\;\mathrm{the}\;\mathrm{Lawton}\;\mathrm{IADL}\;\mathrm{scale}\;\mathrm{in}\;\mathrm{the}\;\mathrm{past})‑1.986\times (\mathrm{calculation}\;\mathrm{score}\;\mathrm{of}\;\mathrm{the}\;\mathrm{MMSE})]\}}$$
where the MoCA score = 0, 1, …, or 30, food preparation score of the Lawton IADL scale in the past = 0, or 1, and calculation score of the MMSE = 0, 1, …, or 5.

Next, logistic regression analysis of dementia *versus* MCI in the subjects with MCI or dementia (*n* = 67) revealed that after the keen competitions during the stepwise variable selection procedure, the orientation score of the Clinical Dementia Rating scale stayed in the final logistic regression model as the most important statistically significant predictor (Table 4). Both the estimated area under the ROC curve = 0.984 and the modified Hosmer and Lemeshow goodness-of-fit *F* test *p* = 0.7378 (*df* = 9, 57) also indicated an excellent fit.

**Table 4:** Multivariate analysis of the predictors of dementia *versus* MCI among the subjects with MCI or dementia by fitting multiple logistic regression model with the stepwise variable selection method.

Covariate	Estimated Regression Coefficient	Estimated Standard Error	*z* Value	*p* Value	Estimated Odds Ratio	95% C.I. of Odds Ratio
Intercept	-8.593	2.286	-3.76	0.0002	–	–
Orientation score of the CDR (0, 0.5, 1, 2, 3)	10.993	2.911	3.78	0.0002	59483.5178	197.852–17883522.326

**Abbreviations:** CDR, Clinical Dementia Rating scale; MCI, Mild cognitive impairment; and 95% C.I., 95% Confidence Interval.

**Goodness-of-fit assessment:** *n* = 67, the estimated area under the Receiver Operating Characteristic (ROC) curve = 0.984 > 0.7, and the modified Hosmer and Lemeshow goodness-of-fit *F* test *p* = 0.7378 > 0.05 (*df* = 9, 57), which indicated an excellent fit.

**Prediction:** To calculate the estimated probability of being dementia (i.e., the *predicted value*, ${\hat{P}}_{i}$) among the subjects with MCI or dementia given the observed covariate values, we can use the following formula. According to the above fitted multiple logistic regression model
$$\mathrm{logit}\left({\hat{P}}_{i}\right)=\log\left(\frac{{\hat{P}}_{i}}{1‑{\hat{P}}_{i}}\right)=‑8.593+10.993\times \left(\mathrm{orientation}\;\mathrm{score}\;\mathrm{of}\;\mathrm{the}\;\mathrm{CDR}\right)$$
the predicted value of observation *i* is
$${\hat{P}}_{i}=\frac{1}{1+\exp\left\{‑\left[‑8.593+10.993\times \left(\mathrm{orientation}\;\mathrm{score}\;\mathrm{of}\;\mathrm{the}\;\mathrm{CDR}\right)\right]\right\}}$$
where the orientation score of the CDR = 0, 0.5, 1, 2, or 3.

### Floor and ceiling effects

**Fig 1:**
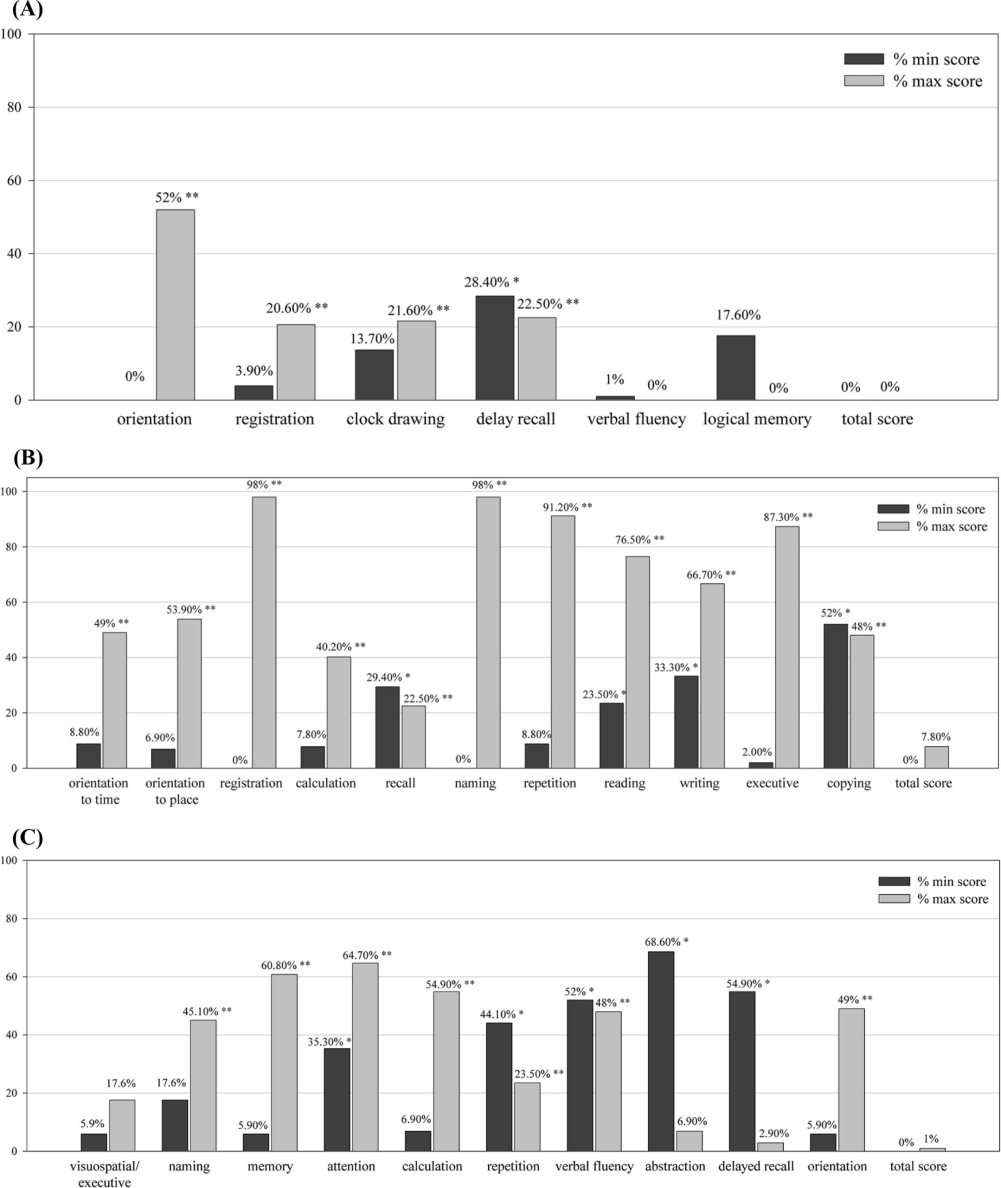
**Percentage of minimum and maximum scores on total and subtests of the (A) Q*mci*-TW, (B) MMSE, and (C) MoCA.** * ≥ 20% of patients obtained minimum scores. ** ≥ 20% of patients obtained maximum scores.

Fig 1 displays percentages of minimum and maximum total and subtest scores on the Q*mci*-TW, MMSE, and MoCA. The total Q*mci*-TW, MMSE and MoCA scores exhibited no floor or ceiling effects. Significant floor effects were identified in 1 (16.7%) of 6 Q*mci*-TW subtests, 4 (36.4%) of 11 MMSE subtests, and 5 (50%) of 10 MoCA subtests, whereas significant ceiling effects were observed in 4 (66.7%) of 6 Q*mci*-TW subtests, all 11 (100%) MMSE subtests, and 7 (70%) of 10 MoCA subtests.

### Psychometric properties

#### Internal consistency

The internal consistency of the Q*mci*-TW screen was good, with a Cronbach’s *α* of 0.85, and the item-to-total correlation were questionable to good, with Cronbach’s *α* of 0.67–0.80. Significant and positive strong correlations (*r* = 0.53–0.76, all *p*’s < 0.001) were found for each of the two Q*mci*-TW screen subtests. Results of internal consistency and inter-item correlation of the Q*mci*-TW screen are presented in Table 5.

**Table 5:** Results of internal consistency and the inter-item correlation of the Q*mci*-TW.

Variable	Cronbach’s α	1.	2.	3.	4.	5.	6.
**Q*mci*-TW**	0.85						
**1. orientation**	0.79	‒					
**2. registration**	0.72	0.63**	‒				
**3. clock drawing**	0.77	0.70**	0.61**	‒			
**4. delayed recall**	0.80	0.76**	0.62**	0.71**	‒		
**5. verbal fluency**	0.80	0.69**	0.71**	0.67**	0.71**	‒	
**6. logical memory**	0.67	0.53**	0.57**	0.59**	0.60**	0.66**	‒

Abbreviations: Q*mci*-TW, Taiwan version of the Quick Mild Cognitive Impairment screen.

*p < 0.05.

**p < 0.001.

#### Test–retest reliability and interrater reliability

We observed ICCs [95% Confidence Intervals (CIs)] of 0.87 (0.67–0.95) and 1.00 (1.00–1.00) with 21 participants for test–retest and interrater reliability, respectively (both *p* < 0.001), suggesting excellent reliability for the Q*mci*-TW.

### Validity

#### Concurrent and construct validity

The correlation of the Q*mci*-TW with the MoCA and MMSE for concurrent validity was positive and very strong (*ρ* = 0.93 and 0.91, respectively; both *p* < 0.001). The correlation of the Q*mci*-TW was positive and moderate with the Barthel Index (*ρ* = 0.46) and strong with the Lawton Instrumental Activities of Daily Living scale (*ρ* = 0.67) for construct validity, respectively (both *p* < 0.001).

### Diagnostic properties

#### Sensitivity, specificity, and predictive values

**Fig 2:**
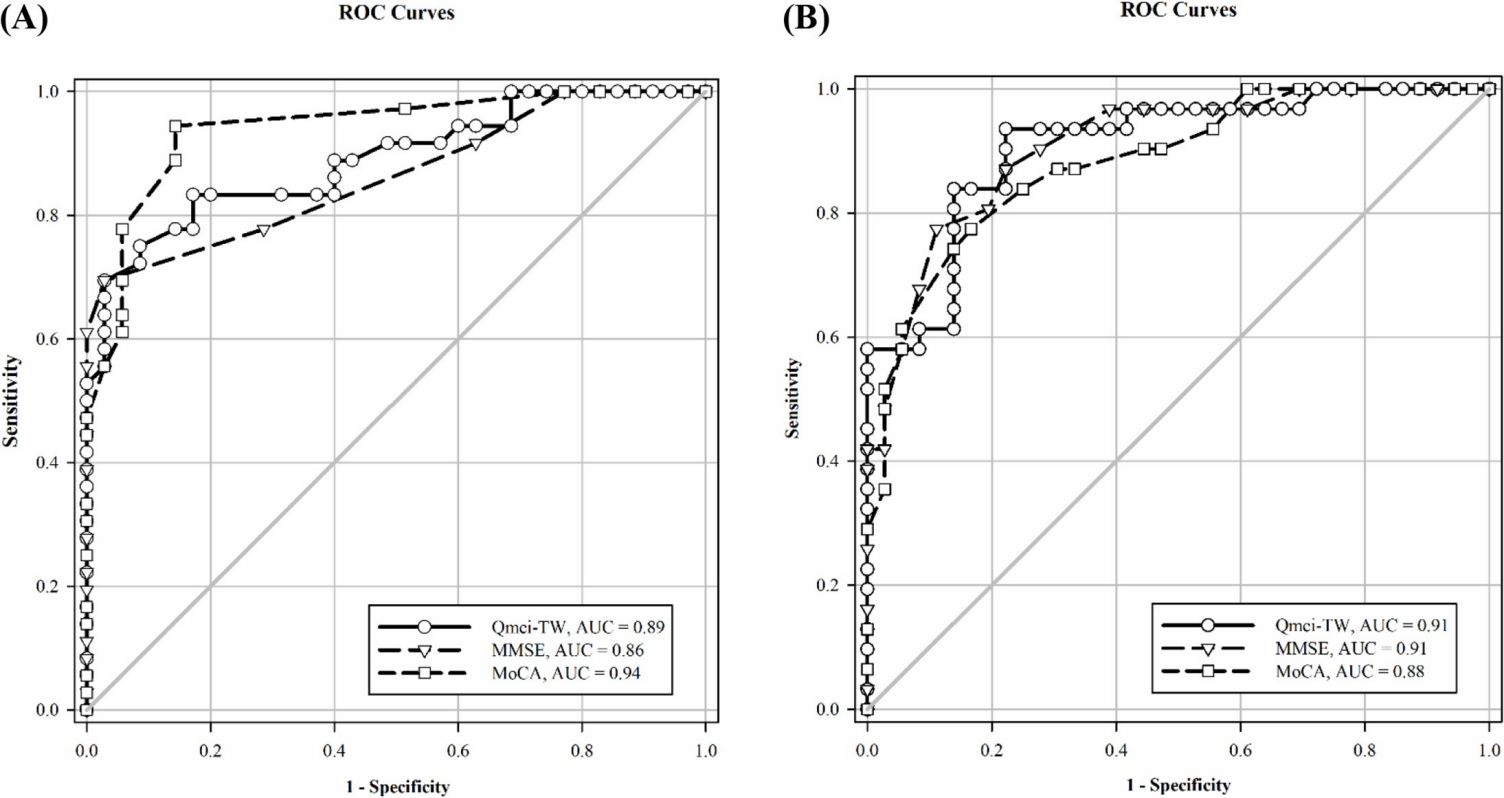
The ROC curves of the Q*mci*-TW, MoCA, and MMSE for differentiating (A) MCI from NC, and (B) dementia from MCI.

Fig 2 illustrates the ROC curves of the Q*mci*-TW, MoCA, and MMSE for differentiating MCI from NC and dementia from MCI. The optimal cut-off scores on the Q*mci*-TW, MoCA, and MMSE for differentiating the participants with MCI from NCs were ≤ 51.5/100 [AUC_Q*mci*-TW_ (95% CI) = 0.89 (0.81–0.97), sensitivity_Q*mci*-TW_ = 69%, specificity_Q*mci*-TW_ = 97%, PPV_Q*mci*-TW_ = 96%, NPV_Q*mci*-TW_ = 76%], ≤ 23/30 [AUC_MoCA_ (95% CI) = 0.94 (0.89–0.99), sensitivity_MoCA_ = 94%, specificity_MoCA_ = 85%, PPV_MoCA_ = 92%, NPV_MoCA_ = 94%], and ≤ 26/30 [AUC_MMSE_ (95% CI) = 0.86 (0.78–0.95), sensitivity_MMSE_ = 69%, specificity_MMSE_ = 97%, PPV_MMSE_ = 96%, NPV_MMSE_ = 76%], respectively. Thus, the Q*mci*-TW exhibited higher and lower accuracy for differentiating the participants with MCI from NCs than did the MMSE (*χ*^*2*^ = 0.54, *p* = 0.46), and MoCA (*χ*^*2*^ = -2.78, *p* = 0.10), respectively. In addition, the MoCA was more accurate in differentiating the participants with MCI from NCs than was the MMSE (*χ*^*2*^ = -3.15, *p* = 0.08). The optimal cut-off scores on the Q*mci*-TW, MoCA, and MMSE for differentiating the participants with MCI from those with dementia were ≤ 31/100 [AUC_Q*mci*-TW_ (95% CI) = 0.91 (0.84–0.98), sensitivity_Q*mci*-TW_ = 94%, specificity_Q*mci*-TW_ = 78%, PPV_Q*mci*-TW_ = 78%, NPV_Q*mci*-TW_ = 93%], ≤ 11/30 [AUC_MoCA_ (95% CI) = 0.88 (0.80–0.96), sensitivity_MoCA_ = 77%, specificity_MoCA_ = 84%, PPV_MoCA_ = 80%, NPV_MoCA_ = 81%], and ≤ 18/30 [AUC_MMSE_ (95% CI) = 0.91 (0.84–0.98), sensitivity_MMSE_ = 77%, specificity_MMSE_ = 89%, PPV_MMSE_ = 86%, and NPV_MMSE_ = 82%], respectively. Thus, the accuracy of the Q*mci*-TW in differentiating the participants with MCI from those with dementia was similar to that of the MMSE (*χ*^*2*^ = -0.0001, *p* = 0.97) and higher accuracy than that of the MoCA (*χ*^*2*^ = 0.70, *p* = 0.40). Moreover, the MMSE was more accurate in differentiating the participants with dementia from MCI than was the MoCA (*χ*^*2*^ = 1.90, *p* = 0.16).

## Discussion

This study demonstrated that the Q*mci*-TW is a reliable and valid cognitive screening instrument potentially useful for differentiating among NCs and individuals with MCI and dementia. The Q*mci*-TW exhibited internal consistency, excellent test–retest reliability, and interrater reliability for clinical use. The Q*mci*-TW also exhibited sound concurrent, and construct validity in comparisons with the MoCA and MMSE and with the Barthel Index and Lawton Instrumental Activities of Daily Living scale respectively. In addition, evaluation criteria for the optimal cognitive screening instruments should also be considered, including bandwidth-fidelity tradeoff, culture fairness measure, economic considerations, and scopes of application. The reliability of the Q*mci*-TW with slightly narrow band is still satisfactory, and the Taiwan version of the Q*mci* is also validated without cultural bias in Taiwan population. Moreover, the Q*mci*-TW may not involve the floor and ceiling effects of the other tests for differentiating among NCs and individuals with MCI and dementia. Hence, the Q*mci*-TW may be preferable, particularly in patients with varied levels of cognitive function. The Q*mci*-TW represents the third Q*mci* screen translation, after the Dutch (Q*mci*-D) [22] and Turkish (Q*mci*-TR) versions [20]; the confirmation of its psychometric validity contributes to the growing evidence supporting clinical use of the Q*mci*.

Our results indicate that the MoCA is the most accurate test for differentiating MCI cases from NCs, followed by the Q*mci*-TW, and the MMSE. In the MoCA with high sensitivity, a positive test confirms MCI diagnosis, whereas in the Q*mci*-TW and MMSE with high specificity, a negative test result rules out diagnosis of MCI. In addition, the Q*mci*-TW and MMSE were found to be more accurate than the MoCA in differentiating dementia cases from MCI cases. In the Q*mci*-TW with high sensitivity, a positive test confirms the diagnosis of dementia, whereas in the MoCA and MMSE with high specificity, a negative test rules out diagnosis of dementia. According to these results, we recommend the use of the MoCA and Q*mci*-TW for detecting MCI and dementia, respectively.

As listed in Table 1, our univariate analyses indicated that age, educational level, and the Geriatric Depression Scale-Short Form score differed significantly among the NC, MCI, and dementia groups. Specifically, older age, lower educational level, and higher incidence of depression were more likely observed in the dementia group. These findings are similar to those of a previous study, which demonstrated that depressed mood, illiteracy, and older age were associated with dementia [42]. Next, multivariate analysis was conducted to assess the partial effects of all the relevant covariates in Tables 1 and 2. As shown in Tables 3 and 4, the lower MoCA score, food preparation score of the Lawton IADL scale in the past, and calculation score of the MMSE, the more likely to be MCI or dementia in all subjects, whereas the higher orientation score of the Clinical Dementia Rating scale, the more likely to be dementia in subjects with MCI or dementia. These findings not only could help us make predictions as a screening tool, but also shed light on the complementary roles of the MoCA, Lawton IADL scale, MMSE, and Clinical Dementia Rating scale. Yet, the Q*mci*-TW did not add much to them in the discriminations among NC, MCI, and dementia.

The current results indicated that the MoCA subtests exhibited the most floor effects, followed by the subtests of the MMSE and Q*mci*-TW. The MMSE subtests exhibited the most ceiling effects, followed by the subtests of the MoCA and Q*mci*-TW. The Q*mci*-TW facilitated accurate evaluation of a wide range of cognition functions with minimal floor and ceiling effects, and thus, was superior to the MoCA and MMSE. In addition, S1 Fig illustrates the ROC curves of the Q*mci*-TW subtests for differentiating MCI from NC and dementia from MCI. The best indicators of the Q*mci*-TW subtests for differentiating the participants with MCI from NCs, and participants with dementia from those with MCI were delayed recall, and orientation, respectively. Our results are similar to those of previous studies demonstrating that orientation is a poorer predictor of MCI with significant ceiling effects [43] and logical memory is a highly sensitive and specific for differentiating the participants with MCI from NCs [33].

In this study, 12.7% of all participants (4 and 9 patients with MCI and dementia, respectively) were illiterate. The optimal cut-off score for differentiating participants with MCI from NCs on the Q*mci*-TW (≤ 51.5/100) was lower than that on the Q*mci* (≤ 60/100) [18, 19], which is potentially attributable to the low levels of education and high levels of illiteracy in Taiwan’s older population. The optimal cut-off score for discriminating participants with MCI from those with dementia on the Q*mci*-TW (≤ 31/100) was much lower than that on the Q*mci*-TR (< 43/100) [20] and the Q*mci*-D (≤ 42/100) [22], explained by the higher proportion of patients with severe dementia in Taiwan population.

This study revealed that patients’ abilities to execute complex instrumental activities of daily living may be a critical factor for differentiating MCI cases from NCs; however, Lawton Instrumental Activities of Daily Living scale scores in the NC group were not significantly higher than those in the MCI group (*p* = 0.07). In the MCI group, all participants maintained instrumental activities of daily living functions related to telephone use, housekeeping, transport mode, and finances; however, with regard to instrumental activities of daily living functions of food preparation, medication responsibility, shopping, and laundry, the number of participants with MCI scoring 0 were 16 (44.4%), 12 (33.3%), 11 (30.6%), and 8 (22.2%), respectively. Maintenance of activities of daily living is a critical factor for distinguishing between individuals with dementia and those with MCI and for differentiating between mild and severe dementia. The Barthel Index scores were significantly higher in the MCI group than in the dementia group (*p* < 0.001), In addition, the Barthel Index scores exhibited a significant negative moderate correlation with Clinical Dementia Rating scale global scores of 1–3 (*ρ* = −0.64, *p* < 0.001).

The key contribution of this study provides that the Q*mci-*TW with satisfactory psychometric and diagnostic properties is a useful and brief cognitive screening instrument to differentiate NCs, MCI and dementia. Notably, logical memory subtest only in the Q*mci-*TW, not in the MMSE, and MoCA, plays an important role for discriminating MCI from NC. Moreover, the Q*mci-*TW with time-limited for answering each questions can enhance the discriminating ability to detect patients with MCI and dementia by taking the response speed into consideration [44] and save much time for clinical practitioners. For early detection and treatment of patients with MCI and dementia, we recommend the use of the Q*mci*-TW in busy clinical settings.

This study has several limitations. First, the potential confounders of age and education may have caused the differences in the AUCs. This limitation is associated with the inherent challenges of a cross-sectional study design, which does not allow for adequate matching between groups. Second, the studied sample size was small and lacked statistical power for use in evaluating accuracy within each participant group. Even though sample size is one of the limitations, the statistically significant findings still deserve our attention, but the inference of the statistically non-significant findings should be conservative due to lack of power. Third, participants with active depression, who may exhibit slower reaction times and processing speeds [45], and patients with dementia subtypes, including frontotemporal dementia, Parkinson’s disease, and Lewy Body dementia, and different MCI syndromes were excluded from this study. These exclusions may have led to some spectrum bias; consequently, our results may not be generalizable to other types of MCI and dementia. Fourth, because of the conceptual constructs of the Q*mci*-TW, MoCA, and MMSE with non-identical cognitive domains, numbers of items, and scoring criteria, the results of the pure inter-correlation analysis were needed to be carefully interpreted [46].

In conclusion, the Q*mci*-TW is a reliable and valid cognitive screening instrument with accurate diagnostic properties for detecting MCI and dementia in Taiwanese individuals. Further research including age- and education-matched NCs, larger sample sizes, younger adults, and other settings, such as psychiatry, and general practice clinics, are required. Nevertheless, this study provides evidence that the Q*mci* and Q*mci*-TW are useful for cognitive screening in clinical practice.

## Supporting information

S1 Table The overview of the Q*mci*, MMSE, and MoCA. (DOCX)

S1 Fig The ROC curves of the Q*mci*-TW subtests for differentiating (A) MCI from NC, and (B) dementia from MCI.
(TIF)
