# Supplementary material for: Psychometric and diagnostic properties of the Taiwan version of the Quick Mild Cognitive Impairment screen
Source: PLoS One. 2018 Dec 3;13(12):e0207851. doi: 10.1371/journal.pone.0207851 (PMC6277119; doi:10.1371/journal.pone.0207851)
Supplement: S1 Table — (DOCX) [file pone.0207851.s001.docx]

**S1 Table. The overview of the Q*mci*, MMSE, and MoCA**

| **Characteristics** | **Q*mci*** | **MMSE** | **MoCA** |
| --- | --- | --- | --- |
| **Author (year)** | Molloy et al. (2012) | Folstein et al. (1975) | Nasreddine et al. (2005) |
| **Administered time** | < 5 minutes. | 5-8 minutes for NCs.  ≥ 15 minutes for dementia. | 15 minutes. |
| **Cognitive domain (item)** | **4 domains (6 items):**  1. Orientation (orientation); 2. Memory (registration, delayed recall, logical memory); 3. Executive function (verbal fluency); 4. Visuospatial function (clock drawing). | **5 domains (11 items):**  1. Orientation (orientation to time, orientation to place); 2. Attention (attention and calculation); 3. Language (naming, repetition, comprehension, Reading, writing); 4. Memory (registration, recall); 5. Visuospatial function (drawing). | **6 domains (12 items):**  1. Orientation (orientation); 2. Attention (digit span, serial 7 subtractions); 3. Language (naming, sentence repetition); 4. Memory (delayed recall); 5. Executive function (modified TMT, tapping test, fluency, abstraction); 6. Visuospatial function (CDT). |
| **Internal consistency** | ‒ | 0.68-0.96 | 0.72-0.87 |
| **Test–retest reliability** | 0.86-0.92 | 0.74-0.99 | 0.86-0.96 |
| **Inter-rater reliability** | 0.90 | 0.83-0.95 | 0.87-0.95 |

**Abbreviations:** Q*mci*, Quick Mild Cognitive Impairment screen; MMSE, Mini-Mental State Examination; MoCA, Montreal Cognitive Assessment; TMT, Trail Making Test; and CDT, Clock Drawing Test.
