# Supplementary figures and images for: Psychometric and diagnostic properties of the Taiwan version of the Quick Mild Cognitive Impairment screen
Source: PLoS One. 2018 Dec 3;13(12):e0207851. doi: 10.1371/journal.pone.0207851 (PMC6277119; doi:10.1371/journal.pone.0207851)

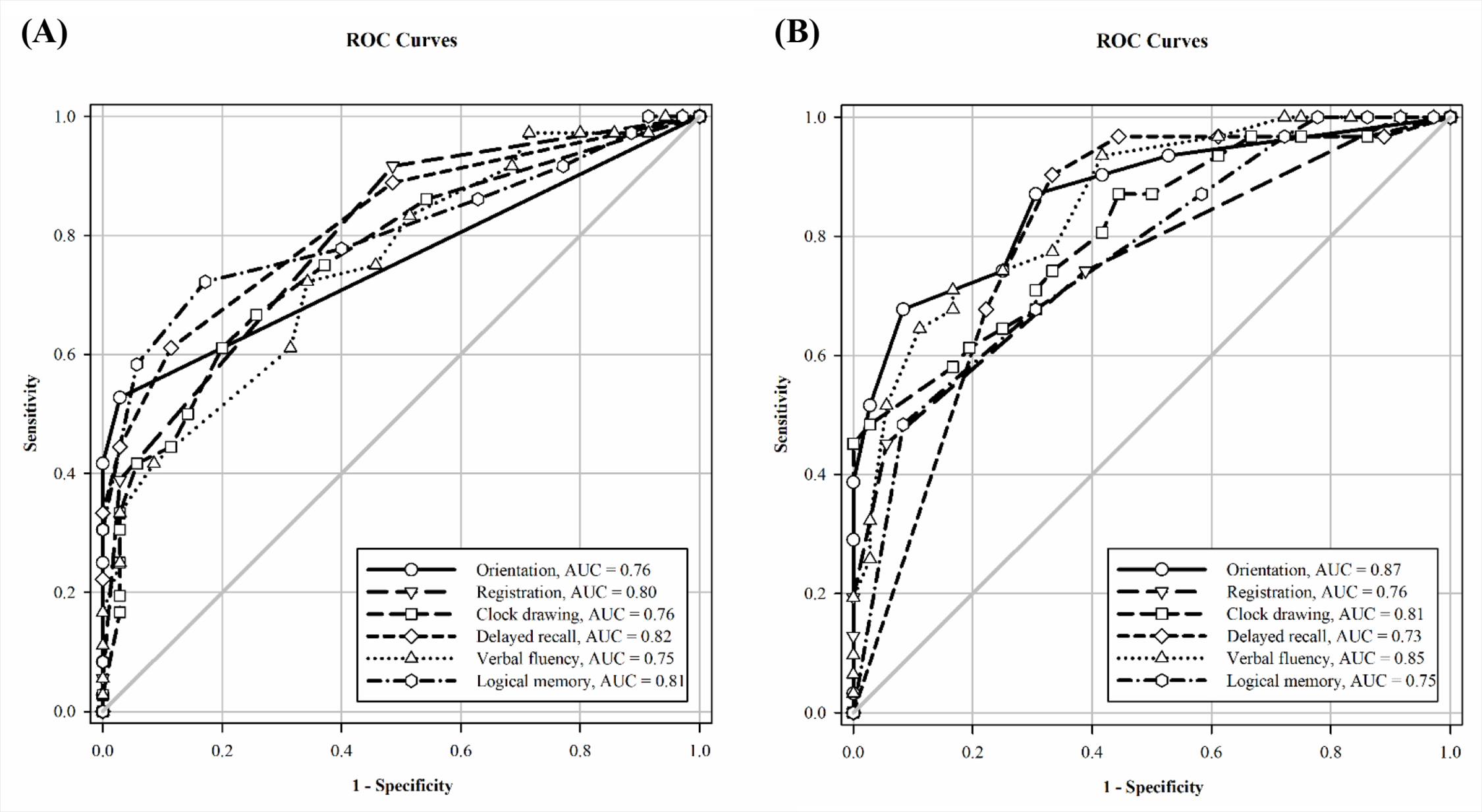

Supplement: S1 Fig — The ROC curves of the Qmci-TW subtests for differentiating (A) MCI from NC, and (B) dementia from MCI. (TIF) [file pone.0207851.s002.tif]
